# Effect of liver transplantation on intestinal permeability and correlation with infection episodes

**DOI:** 10.1371/journal.pone.0235359

**Published:** 2020-06-26

**Authors:** Francesca Romana Ponziani, Venanzio Valenza, Erida Nure, Giuseppe Bianco, Giuseppe Marrone, Antonio Grieco, Maurizio Pompili, Antonio Gasbarrini, Salvatore Agnes, Gabriele Sganga

**Affiliations:** 1 Internal Medicine, Gastroenterology, Hepatology—Fondazione Policlinico Universitario Agostino Gemelli IRCCS, Catholic University, Rome, Italy; 2 Image Diagnostics, Oncological Radiotherapy and Hematology Sciences—Fondazione Policlinico Universitario Agostino Gemelli IRCCS, Catholic University, Rome, Italy; 3 General Surgery and Liver Transplantation—Fondazione Policlinico Universitario Agostino Gemelli IRCCS, Catholic University, Rome, Italy; 4 Emergency Surgery—Fondazione Policlinico Universitario Agostino Gemelli IRCCS, Rome, Italy; Texas A&M University, UNITED STATES

## Abstract

**Background:**

Liver cirrhosis has been known to be associated with increased intestinal permeability (IP); however, little is known about the modification of IP after liver transplantation (LT). The present study was aimed to assess IP after LT and evaluated its association with laboratory tests and clinical parameters, as well as with the development of infections.

**Methods:**

LT recipients were consecutively enrolled and compared with an equal number of patients with liver cirrhosis and healthy subjects. IP was assessed by urinary excretion of chromium-51 ethylenediaminetetraacetic acid (^51^Cr-EDTA).

**Results:**

The median ^51^Cr-EDTA excretion was found to be higher in 35 LT recipients as compared with that in the healthy controls [4.77% (2.79–6.03) vs. 2.07% (1.57–2.42), p<0.0001], and comparable to that in the cirrhotic patients [3.69% (2.34–6.57), p = 0.445]. ^51^Cr-EDTA excretion was not associated with clinical variables, the type of immunosuppressive therapy, donor-related factors, comorbidities and incidence of infections [infection/no infection: 4.97% (3.14–7.03) vs 4.62% (2.79–5.82), p = 0.938].

**Conclusion:**

LT recipients show an increased IP, similar to that in patients with liver cirrhosis. However, it is not associated with a high risk of infections. Further investigations into the pathogenesis of this persistent impairment of the intestinal barrier are warranted.

## Introduction

Liver cirrhosis has been reported to be associated with an increase in the intestinal permeability (IP) [1]. Portal hypertension, immune response alteration and changes in the gut microbiota result in the dysfunction of the intestinal barrier, which worsens with the progression of liver disease [2].

Increased IP is associated with bacterial translocation and systemic inflammation, which together play a crucial role in the development of liver disease complications, such as hyperdynamic circulation, hepatic encephalopathy, spontaneous bacterial peritonitis, and hepatorenal syndrome [3]. These manifestations exert clear prognostic significance, as evident from an increased risk of mortality [4].

Liver transplantation (LT) is the most effective treatment for end-stage liver disease and associated complications [5]. As a change in IP is a hallmark of liver cirrhosis, it is reasonable to hypothesize that replacing the cirrhotic liver could resolve the dysfunction of the gut-liver axis as well. Indeed, recent studies have clearly shown that LT could exert a beneficial effect on gut dysbiosis in patients with liver cirrhosis. Moreover, these studies demonstrated an improvement in endotoxemia as well as in ammonia, bile acids, lipidomic and metabolomic profiles [6, 7]. However, it is currently unknown whether LT could also lead to a complete recovery of altered IP, which is commonly found in patients with liver cirrhosis.

The present study investigated the IP in LT recipients and evaluated its possible correlation with laboratory tests, immunosuppressive treatment and post-LT clinical outcomes.

## Materials and methods

Due to difficulties in carrying out a longitudinal enrollment in the population of cirrhotic patients before and after LT, we decided perform a study with cross-sectional design. Patients who had previously undergone LT and consecutively evaluated at the Liver Transplant inpatient and outpatient clinics of the Fondazione Policlinico Agostino Gemelli IRCCS in Rome were included in the study (recruitment period: January 2011-December 2015). Age-matched cirrhotic patients (13 Child-Pugh score A, 11 Child-Pugh score B and 11 Child-Pugh score C) and healthy individuals, selected among the patients’ family members and the medical staff, served as control groups.

For all the subjects included in the study, the following exclusion criteria were adopted: treatment with antibiotics (including rifaximin), probiotics, prebiotics, proton pump inhibitors and laxatives during the last month; diseases involving the gut (e.g., inflammatory bowel disease, celiac disease); significant alcohol consumption in the last six months (>21 standard drinks on an average per week in men and >14 standard drinks on an average per week in women). Furthermore, LT recipients showing the signs of cirrhosis according to histological and/or clinical findings (laboratory parameters, ultrasound findings, portal hypertension at liver imaging or endoscopy) were excluded.

The IP was assessed by performing the chromium-51 ethylenediaminetetraacetic acid (^51^Cr-EDTA) assay. All LT recipients were also subjected to laboratory examinations as well, including liver function tests [serum creatinine, alanine transaminase (ALT), gamma-glutamyl transferase (GGT), total bilirubin, albumin, platelet (PLT) count and international normalized ratio (INR)]; body mass index (BMI), type of immunosuppressive drug regimen, Child-Pugh and model for end-stage liver disease (MELD) scores before LT, alcohol intake before LT, and previous history of hepatocellular carcinoma (HCC) were also recorded. Follow up data concerning the occurrence of infections (within 6 months since IP assessment) were registered.

The study was approved by the Institutional Review Board of the Fondazione Policlinico Agostino Gemelli IRCCS (protocol ID 741) and was conducted in accordance with the principles of the Declaration of Helsinki. All patients gave their written informed consent to participate in this research protocol.

### Evaluation of IP

IP was assessed using ^51^Cr-EDTA assay, as described by Bjarnason and Peters [8], with only minor modifications.

After an overnight fast, a test solution of 0.37 MBq (10 μCi) of ^51^Cr-EDTA diluted in 10 mL of tap water was given to each subject. Urine was collected for the next 24 hours. Food and drinks were allowed ad libitum 30 min later with the exception of coffee, tea and alcoholics.

Two samples (3 mL) of the pooled 24-hour urine collection were counted, in a gamma counter system (Perkin Elmer; Wizard 3, 1480 automatic gamma counter), together with a 3 mL sample of a 1/50th of the orally administered dose. The estimated radiation dose received during the test was <0.01 mSieverts (effective dose equivalent), which is within the variations of natural background radiation [8, 9].

The absorption of ^51^Cr-EDTA was expressed as a percentage of the orally administered dose that was excreted in the urine during 24 hours, using the following formula: [(average urinary count x urinary volume) x (standard counts x 50)] - 1.

The passage of high molecular weight ^51^Cr-EDTA into the bloodstream is directly related to the level of IP and is poorly affected by bacterial degradation in the case of small intestinal bacterial overgrowth. In normal subjects, 1% to 3% of the orally administered dose of ^51^Cr-EDTA gets absorbed from the gastrointestinal tract [10].

### Statistical analysis

Owing to the non-normal distribution of data, statistical analysis was performed using non-parametric tests, with R statistics program (version 3.4.0). Categorical variables were expressed as frequency and percentage, continuous ones as median and interquartile range.

^51^Cr-EDTA excretion was compared among LT recipients, cirrhotic patients and healthy controls. Within the LT group, any difference in IP according to gender, Child-Pugh class or significant alcohol intake before LT, previous history of HCC, type of immunosuppressive drug, steatosis of the graft, comorbidities or occurrence of infections was evaluated using the Wilcoxon test and Kruskal-Wallis test with Dunn post-hoc analysis. Differences according to the Child-Pugh class were also tested in controls with liver cirrhosis. The correlation between 51Cr-EDTA excretion and age, BMI, MELD score before LT, donor age or laboratory parameters in the LT group, as well as MELD score or PLT count in the group of cirrhotic controls was also performed using the Spearman’s test. Differences between the groups were considered significant for p-values <0.05.

## Results

Among 79 consecutively evaluated LT recipients, 44 were excluded (16 were taking drugs potentially altering IP, 14 refused to participate in the study, 12 presented signs of infection, 2 where affected by inflammatory bowel disease), whereas 35 were considered eligible for the study. Demographic and clinical features of the study population including cirrhotic and healthy controls are described in Table 1.

**Table 1:** Demographic and clinical characteristics of liver transplant (LT) recipients and cirrhotic controls. Continuous variables are presented as median (IQR), categorical ones as frequency (%).

VARIABLE	LT	CIRRHOTIC	HEALTHY	p-value	p-value	p-value
	RECIPIENTS	CONTROLS	CONTROLS	LT vs CIRRHOSIS	LT vs CONTROLS	CIRHOSIS vs CONTROLS
	(35)	(35)	(35)			
***Age (years)***	55	61.5	54.5	0.14	0.93	0.10
	(48.5–60.5)	(52.5–67)	(44.75–65)			
***Gender (M/F)***	27	27	27	-	-	-
	(77.14) /8 (22.85)	(77.14) / 8 (22.85)	(77.14) / 8 (22.85)			
***BMI (kg/m^2)***	24.39	25.82	-	0.07	0.24	0.48
	(21.84–26.1)	(20.33–32.72)				
***Etiology***			-	-	-	-
• ***viral***	20 (57.14)	21 (60)	-	-	-	-
• ***alcohol***	7 (20)	7 (20)	-	-	-	-
• ***NAFLD***	6 (17.14)	7 (20)	-	-	-	-
• ***PSC***	1 (2.85)	-	-	-	-	-
• ***hemochromatosis***	1 (2.85)	-				
***Child-Pugh***	4 (11.42) / 10 (28.57) / 21 (60)*	13 (37.14) / 11 (31.42) / 11 (31.42)	-	-	-	-
***(A/B/C)***						
***MELD***	15	13	-	-	-	-
	(11–27.5)*	(9.5–20)				
***HCC***	12	9	-	-	-	-
	(34.28)*	(25.71)				
***Immunosuppressive drug regimen***						
• ***cyclosporine***	8 (22.85)	-	-	-	-	-
• ***tacrolimus***	21 (60)	-	-	-	-	-
• ***everolimus***	6 (17.14)	-	-	-	-	-
***Comorbidities***	25 (71.42)	-	-	-	-	-
• ***hypertension***	10 (28.57)	-	-	-	-	-
• ***diabetes***	9 (25.71)	-	-	-	-	-
• ***dyslipidemia***	3 (8.57)	-	-	-	-	-
• ***cardiovascular***	5 (14.28)	-	-	-	-	-
***Donor age (years)***	48 (30.75–62)	-	-	-	-	-
***Graft fatty infiltration***						
• ***< 5%***	17 (48.57)	-	-	-	-	-
• ***5–33%***	18 (51.43)	-	-	-	-	-
***Time from LT (months)***	6.6 (1.2–32.8)	-	-	-	-	-
***Creatinine (mg/dL)***	1	1	0.87	0.81	**0.01**	**0.01**
	(0.85–1.2)	(0.79–1.45)	(0.74–1)			
***ALT (IU/L)***	37	26	19	0.83	**0.002**	**0.004**
	(17.5–84)	(21–55)	(17–23.25)			
***GGT (IU/L)***	100	73	20	0.70	**<0.0001**	**<0.0001**
	(29–236.5)	(39–122)	(15–24)			
***Alkaline phosphatase (IU/L)***	265	112	73	**0.002**	**<0.0001**	**<0.0001**
	(146.5–559.5)	(79.5–255.5)	(68–79.25)			
***Total bilirubin (mg/dL)***	1.1	2.1	0.7	**0.0004**	**0.0002**	**<0.0001**
	(0.73–1.91)	(1.35–3.65)	(0.5–0.8)			
***Albumin (g/dL)***	4	3.4	4.1	**<0.0001**	**0.27**	**<0.0001**
	(3.7–4.3)	(2.7–3.7)	(3.9–4.2)			
***PLT (x 10^9/L)***	151	84	233	**0.0002**	**0.0003**	**<0.0001**
	(98.5–204.2)	(66–106)	(200–255.25)			
***INR***	1.1	1.43	0.99	**<0.0001**	**<0.0001**	**<0.0001**
	(1–1.18)	(1.2–1.65)	(0.98–1)			

Statistically significant comparisons are highlighted in bold.

* before LT

BMI = body mass index; NAFLD = non-alcoholic fatty liver disease; PSC = primary sclerosing cholangitis; LT = liver transplant; MELD = model for end-stage liver disease; HCC = hepatocellular carcinoma; ALT = alanine transaminase; GGT = gamma-glutamyl transferase; PLT = platelet; INR = international normalized ratio

Twenty patients received LT for cirrhosis of viral etiology, 7 for alcoholic liver disease, 5 for non-alcoholic fatty liver disease, 1 for primary sclerosing cholangitis, and 1 for hemocromatosis. Median donor age was 48 (30.75–62) years, mild steatosis (5–33%) was found in 18 grafts at liver biopsy, whereas fatty infiltration was < 5% in the rest of the cases. Primary immunosuppressive therapy included calcineurin inhibitors (29 patients: 8 cyclosporine, 21 tacrolimus) and everolimus (6 patients). In some cases, calcineurin inhibitors were associated with mycophenolate mofetil (5 patients) or everolimus (3 patients). Seventy-one percent of LT recipients presented at least one comorbidity, the most frequent one being hypertension and diabetes.

In the control group with cirrhosis, the etiology of liver disease was predominantly (60%) virus-related, followed by alcoholic and non-alcoholic fatty liver disease (20%). Thirty-seven percent of patients presented a well-compensated liver disease, whereas 31% were classified as Child-Pugh B or C. Twelve (34%) LT recipients and 9 cirrhotic patients (25%) had a history of hepatocellular carcinoma. No difference in the body mass index (BMI) was observed between groups.

### Assessment of IP in LT recipients and its correlation with laboratory parameters and clinical outcomes

**Fig 1:**
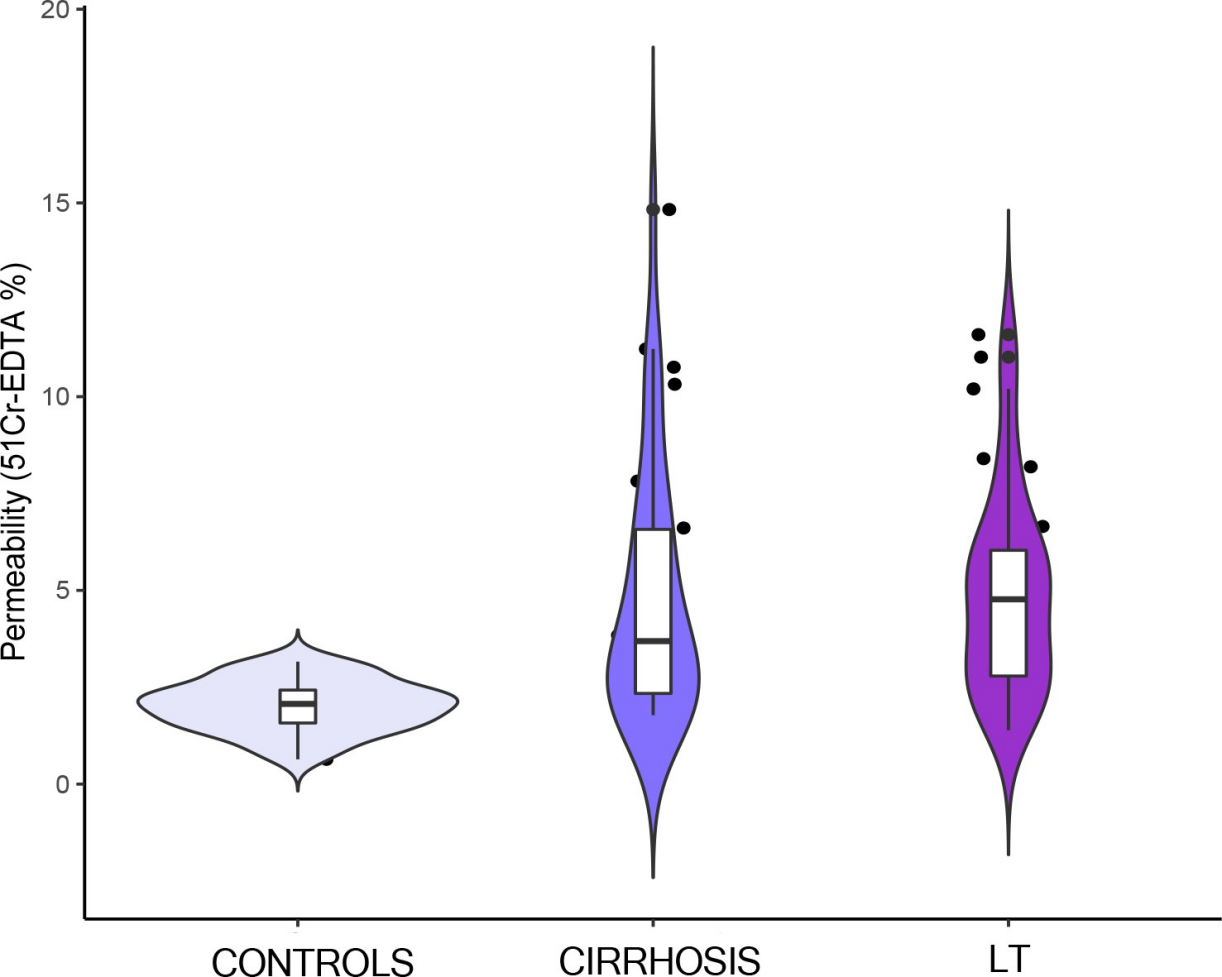
Median ^51^Cr-EDTA excretion in liver transplant (LT) recipients, cirrhotic patients and healthy controls. Boxes represent the first quartile, median, and the third quartile, kernel density plots show the distribution shape of the data.

The median ^51^Cr-EDTA excretion was found to be 4.77% (2.79–6.03) in the LT group (Fig 1).

It was higher than that reported in the healthy controls [2.07% (1.57–2.42), p<0.0001], but similar to that in the cirrhotic patients [3.69% (2.34–6.57), p = 0.44]. In the subgroup of cirrhotic patients, ^51^Cr-EDTA excretion showed a trend towards increase with the worsening of liver function and an inverse correlation with markers of portal hypertension [Child class: A 2.77% (2.1–7.82), B 3.84% (2.52–5.91), C 4.78% (3.11–6.21), p = 0.80; correlation with MELD score 0.237, p = 0.17; correlation with PLT count -0.232, p = 0.18].

**Fig 2:**
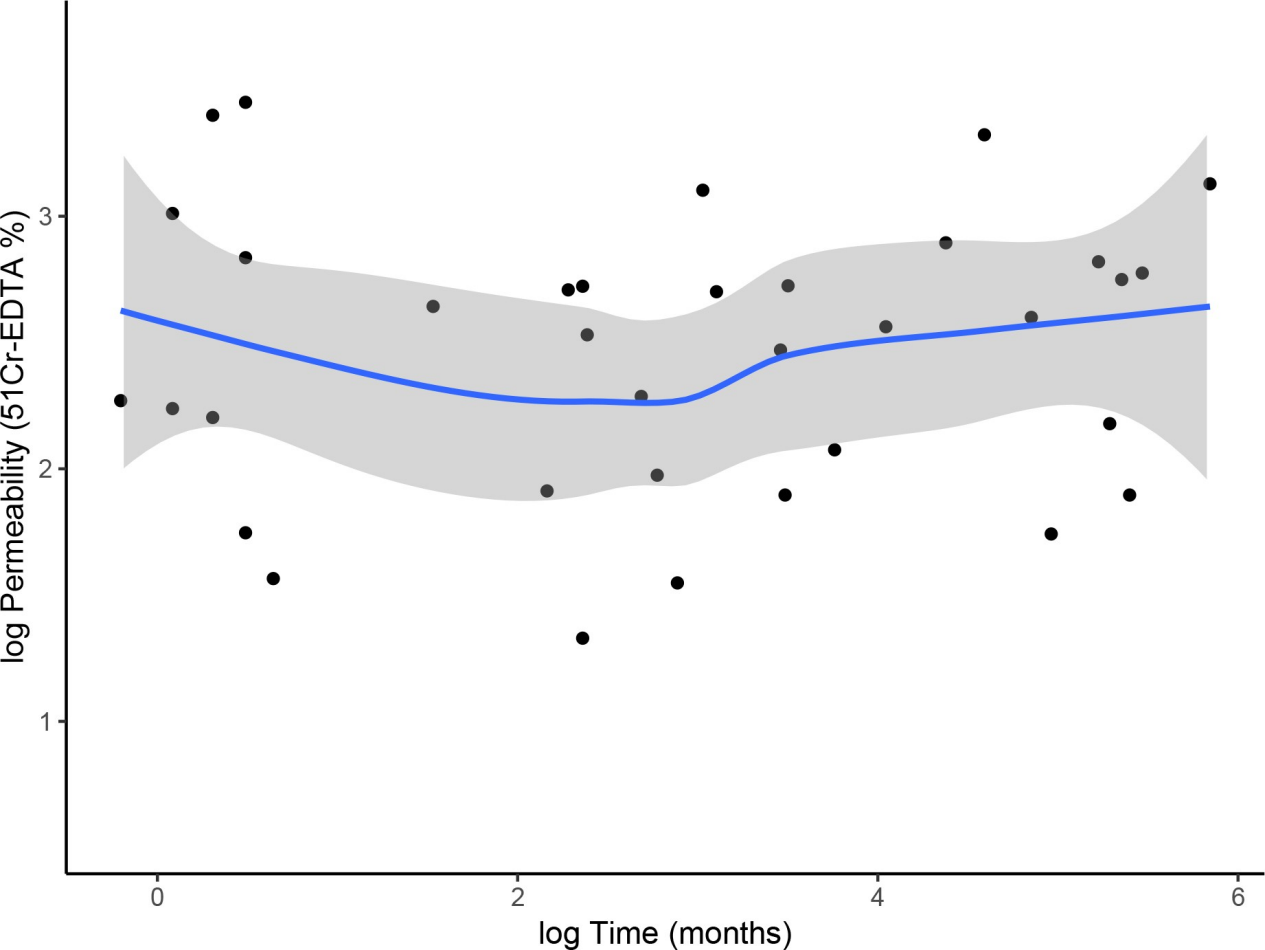
Scatter diagram representing the relationship between ^51^Cr-EDTA excretion (%) and time (months) since liver transplant (LT). The trend is displayed by the blue fitted line, the confidence intervals by the gray bands. Data are plotted on a logarithmic scale.

We unexpectedly observed that IP was still elevated after LT; therefore, we tried to investigate whether the time elapsed since LT could have influenced this result. IP was measured at a median time of 6.6 (1.2–32.8) months after LT. In particular, 13 patients had undergone LT within three months since IP assessment, 10 within 12 months and 12 for more than 12 months. However, no significant correlation was observed between the time interval since LT and IP (0.07, p = 0.69; Fig 2). This was also confirmed using a cut-off time (3 months) for grouping patients (median permeability: > 3 months 4.77% (2.93–5.9), ≤ 3 months 4.36 (2.7–6.1); p = 0.91).

**Fig 3:**
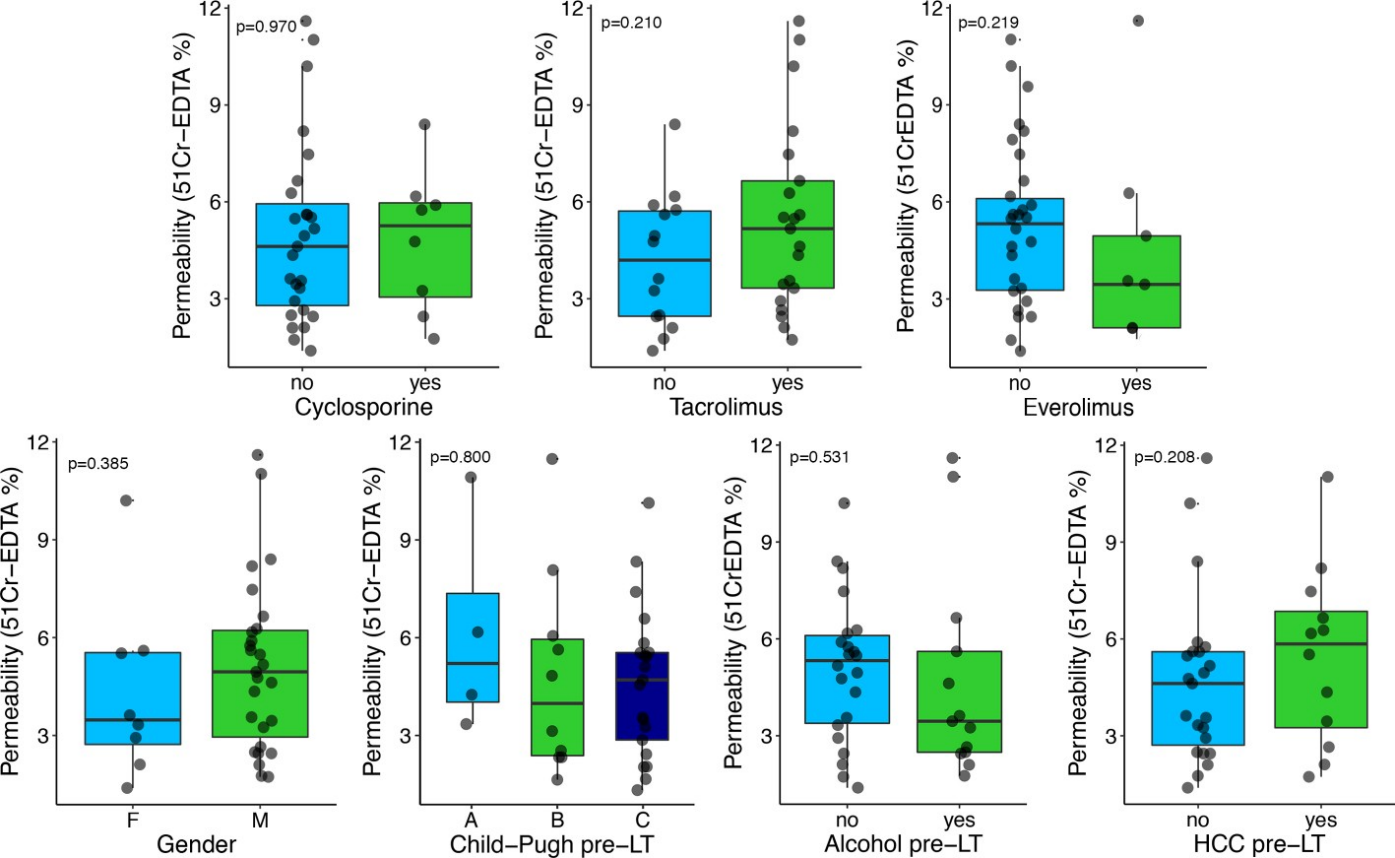
Median ^51^Cr-EDTA excretion in liver transplant (LT) recipients, according to the immunosuppressive drug regimen, gender, Child-Pugh score, alcohol consumption or history of hepatocellular carcinoma (HCC) before LT. Boxes represent the first quartile, median, and the third quartile, while kernel density plots show the distribution shape of the data.

We next explored if any factor could affect IP after LT. We found that ^51^Cr-EDTA excretion was not influenced by the type of immunosuppressive treatment (Fig 3).

**Fig 4:**
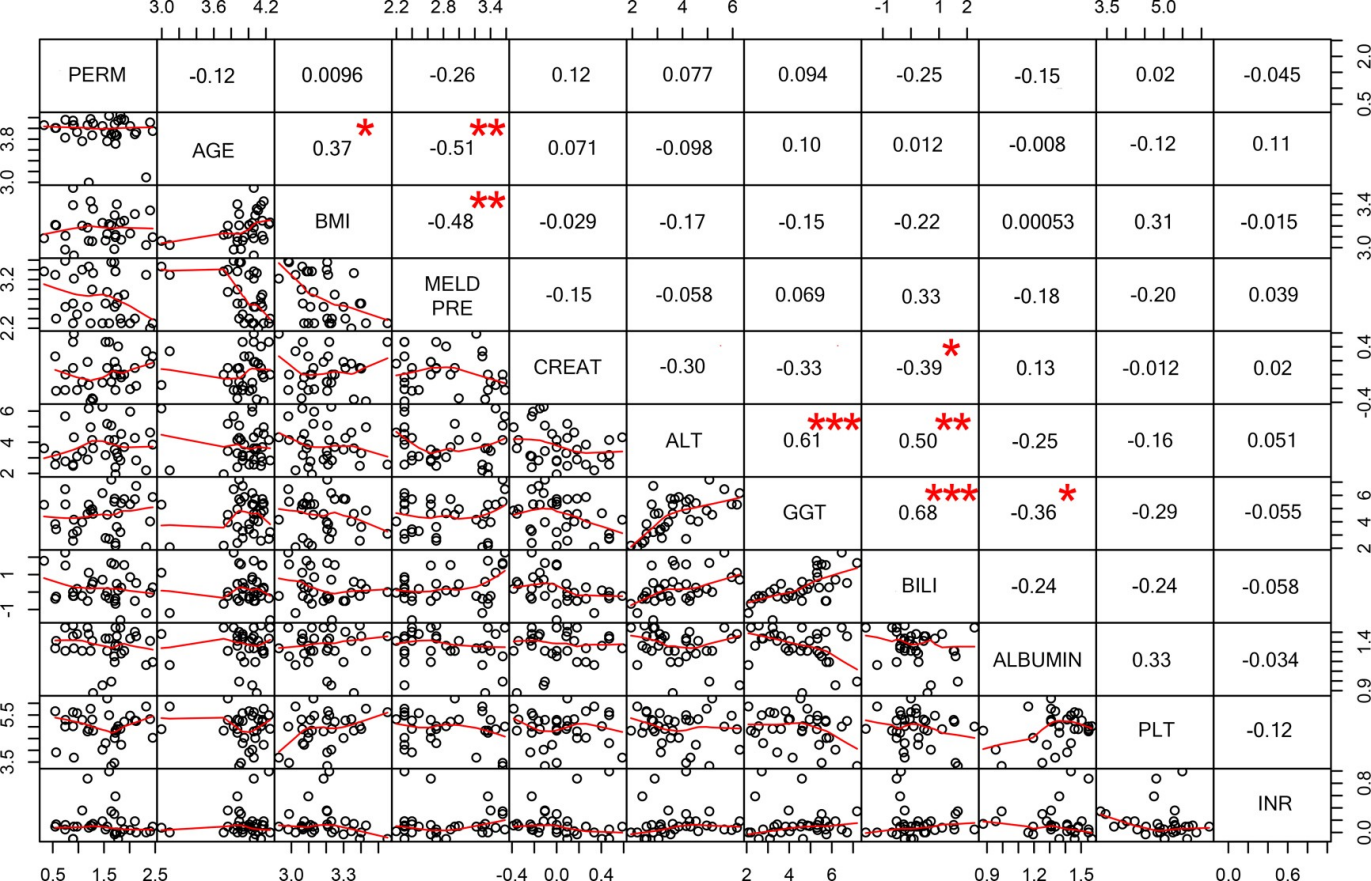
Correlation of demographic, clinical data and intestinal permeability among liver transplant (LT) recipients. The distribution of each variable is shown on the diagonal axis. Scatter plots with the fitted trend line are displayed on the bottom, Spearman’s correlation coefficient is shown at the top region of the image. Significance level is represented by a star: p< = 0.0001 ***, 0.0001<p<0.001 **, 0.001<p<0.01 **, 0.01<p<0.05 *. Data are plotted on a logarithmic scale. PERM = permeability; BMI = body mass index; MELD = model for end-stage liver disease; CREAT = creatinine; ALT = alanine transaminase; GGT = gamma-glutamyl transferase; BILI = bilirubin; PLT = platelet; INR = international normalized ratio.

There was no significant correlation between age, BMI, laboratory parameters or MELD score before LT and ^51^Cr-EDTA excretion, as well as no difference was observed when Child-Pugh score or alcohol intake before LT, gender or previous history of HCC were considered (Figs 3 and 4). We did not observe any association between ^51^Cr-EDTA excretion and LT recipients’ comorbidities (any comorbidity: p = 0.49; hypertension: p = 0.66; diabetes: p = 0.64; dyslipidemia: p = 0.77; cardiovascular: p = 0.95) or donor-related factors such as age (0.195 p = 0.27) or graft steatosis (p = 0.26).

The second aim of the study was to explore the association between IP and infection episodes in the LT group.

Eight patients presented an episode of bacterial infection (cholangitis: 4 cases, sepsis: 3 cases, pneumonia: 1 case). However, we did not observe an increased ^51^Cr-EDTA excretion in these patients [infection vs no infection: 4.97% (3.14–7.03) vs 4.62% (2.79–5.82), p = 0.94].

## Discussion

LT is considered the best treatment for end-stage liver disease; however, at present, its effects on IP have remained unknown. The present study demonstrated that altered IP could not be recovered to normality after LT, irrespective of the time elapsed since LT.

To date, only two small studies [11, 12] have investigated IP in the setting of LT. According to Parrilli et al., IP of LT recipients was comparable to that of healthy subjects, whereas Gabe et al. reported an increased IP and impaired intestinal absorptive capacity.

In both studies, the investigators reported an increased ratio of lactulose and L-rhamnose urinary excretion, a marker of permeability of the small intestine. Furthermore, Parrilli et al. reported a reduced urinary excretion of L-rhamnose, suggesting a selective alteration of transcellular permeation. Moreover, this study group explored the gastroduodenal permeability by measuring the urinary sucrose excretion, which was found to be normal.

Therefore, data available so far are based on the use of sugar probes and reflect the permeability of the proximal part of the gastrointestinal tract. Furthermore, these studies made no comparison with the pre-LT setting, making it difficult to hypothesize the effect of LT on IP.

In the present study, we investigated for the first time the permeability of the small and the large intestine of LT recipients by measuring the urinary excretion of ^51^Cr-EDTA urinary excretion. Indeed, compared to lactulose, L-rhamnose and sucrose, ^51^Cr-EDTA is not metabolized along the gastrointestinal tract and therefore reaches the colon in an unaltered form. We found that LT recipients have an increased IP compared with that in healthy subjects. Interestingly, a comparison of LT recipients with a group of cirrhotic patients, most of whom showing moderate or severe impairment of liver function, revealed no significant difference. Notably, we excluded patients with post-transplant liver cirrhosis, and clinical data highlighted better liver function tests in LT recipients than in cirrhotic patients. Therefore, this finding suggests that the altered IP in patients with liver cirrhosis could not be improved by LT.

To assess the effect of the time interval elapsed since LT on IP, we included in this study patients at different post-LT periods. However, we failed to find any significant correlation between IP and time elapsed since LT. This may reflect an irreversible modification of the gut barrier, which cannot be restored by the improvement in the liver function. Notably, this observation was in line with the results previously obtained in both the early (mean 16 days) [12] and the late (2 to three years) [11] post-LT period. However, our study was the first to analyze together different time points.

Although these results may suggest that the permeability of the whole intestine gets irreversibly damaged by liver cirrhosis and is not improved by LT, it is well-known that LT is considered an effective treatment for portal hypertension, a factor involved in the pathogenesis of increased IP in patients with cirrhosis [13].

Therefore, we further explored possible factors that could be responsible for the observed alteration of IP in LT recipients.

It has been previously demonstrated in rats [14, 15] and humans [11, 12] that tacrolimus and cyclosporine increase the IP and alter the intestinal absorptive capacity by reducing the availability of ATP due to impaired mitochondrial function. However, as already discussed, these studies mainly focused on the small intestinal permeability, with impairment reported in the transcellular permeability pathway over the paracellular one. In light of the above, our study was the first to explore the effect of immunosuppression on the permeability of the whole intestine. Moreover, we also demonstrated that the alteration of IP was independent of the type of immunosuppressive drug regimen. Probably, immunosuppressive drugs impair the permeability of the whole intestine, and this effect seems to be similar for calcineurin and mammalian target of rapamycin (m-TOR) inhibitors.

Among the other investigated factors, we did not find any significant correlation between donor-related factors, comorbidities, clinical and laboratory parameters, alcohol consumption or history of HCC before LT and IP.

Finally, we assessed if the alteration of IP could have a role in increasing the risk of infections in LT recipients. There was no relationship between ^51^Cr-EDTA excretion and occurrence of infections. This result raises several considerations. Although we found a similar increase in the IP in both cirrhotic patients and LT recipients, this did not seem to have any clinical relevance after LT. A similar result was reported by Vogt et al. and Benjamin et al. [16, 17], who failed to demonstrate a link between small intestinal permeability, overall survival, transplant-free and infection-free survival; conversely, markers of inflammation, such as interleukin 6 (IL-6), and of enterocytes death were better predictors of these clinical outcomes [17]. Indeed, the detrimental effects of increased IP-related bacterial translocation in patients with cirrhosis are well-known, being crucial in the development of liver disease complications [1, 18]. The condition gets further worsened by gut dysbiosis, mainly characterized by an increase in potentially pathogenic bacteria and a decrease in autochthonous ones, which is a hallmark of liver cirrhosis [19, 20]. Conversely, it has been recently demonstrated that LT ameliorates gut microbiota dysbiosis and improves its metabolic functions [6, 7]. In conclusion, these findings suggest that increased IP alone is not sufficient to induce clinically significant alterations in LT recipients. Improvement in gut microbial composition and function, as well as the restoration of the normal physiology of the gut-liver axis consequent to LT, may act as protective factors.

The present study had certain limitations. This was a cross-sectional design, as it is difficult to perform a longitudinal study in the LT setting, owing to multiple confounding factors in both the waiting list period and the post-LT period (e.g., surgical and non-surgical procedures, comorbidities, and drugs potentially altering the intestinal barrier) and the need for a long period of follow-up. Furthermore, we included patients at different post-LT periods; however, there was no influence of the time elapsed since LT on IP values, supporting our results to be reliable. Moreover, we selected to measure IP by assessing ^51^Cr-EDTA excretion. This method has already been used to test IP in patients with liver cirrhosis [3]. Although the assay could be easily performed, unfortunately, it is not easily available in the clinical practice. The analysis of serum zonulin and/or lipopolysaccharides (LPS) serum levels as markers of intestinal permeability and bacterial translocation, that could be also quantified in most laboratories, would have increased the accuracy of our results but, unfortunately, it was not included in the original design of the study. Finally, we recognize that the quantification of circulating cytokines would have been important to assess the inflammatory status of LT recipients and could have provided additional information to explain our findings.

In conclusion, LT recipients show an IP comparable to that of patients with liver cirrhosis; however, the lack of IP improvement after LT seems to have poor clinical relevance. Further studies are required to assess the impact of immunosuppressive drugs on IP and the functional implications of our findings, especially with respect to the systemic inflammatory response.
